# Positional training demands in the English Premier League and English Championship. A longitudinal study across consecutive seasons

**DOI:** 10.5114/biolsport.2025.139078

**Published:** 2024-05-07

**Authors:** Ryland Morgans, Ibrahim H. Ceylan, John Radnor, Ben Ryan, Matthew King, Piotr Zmijewski, Rafael Oliveira

**Affiliations:** 1 School of Sport and Health Sciences, Cardiff Metropolitan University, Cardiff, UK; 2 Faculty of Kazim Karabekir Education, Physical Education of Sports Teaching Department, Ataturk University, Erzurum, Turkey; 3 Brentford FC Football Research Centre, Brentford FC, London, UK; 4 Jozef Pilsudski University of Physical Education in Warsaw, 00-809 Warsaw, Poland; 5 Research and Development Center Legia Lab, Legia Warszawa, Poland; 6 Sports Science School of Rio Maior – Instituto Politecnico de Santarem, 2040–413 Rio Maior, Santarém District, Santarém, Portugal; 7 Life Quality Research Centre, 2040–413 Rio Maior, Portugal; 8 Research Center in Sport Sciences, Health Sciences and Human Development, Vila Real, Portuga

**Keywords:** Global Positioning System, Training load, Elite European soccer players, Competitive level, Seasonal trends, Football

## Abstract

The aims of this study were to: compare training loads between the English Premier League (EPL) and English Championship League (ECL) and examine differences between playing positions. Forty-six 1^st^ team players from the same club participated in the study. GPS metrics were obtained during all EPL and ECL training sessions across four consecutive seasons, 2019–20 to 2022–23. The study team was promoted from the ECL at the end of season 2020–21. There was a significant interaction effect between position and league for all GPS metrics (p < 0.001; η^2^ = 0.001–0.003), except for relative high-speed running (HSR) distance, sprint distance, and sprint efforts (p > 0.05). A significant main effect for league for all GPS metrics (p < 0.001; η^2^ = 0.001–0.009) was found, with EPL training sessions resulting in greater total distance per minute, HSR distance per minute, high metabolic load distance (HMLD) per minute, number of HML efforts, accelerations, and decelerations per minute compared to training in the ECL (p < 0.001; d = 0.061–0.224). For position, a significant main effect for all GPS metrics (p < 0.001; η^2^ = 0.001–0.005) was observed. Centre midfielders covered more distance per minute than all other positions (p < 0.001, d = 0.040–0.167). In conclusion, higher training values in the EPL were evident, except for centre forwards, providing some guidance on the differing positional physical demands that may support coaches and practitioners to design position-specific drills incorporating physical and technical/tactical strategies.

## INTRODUCTION

In soccer, various training methods are implemented that produce differing physical stimuli and monitoring these external load demands is widely adopted at different levels due to its significant role in providing neuromuscular stimulation and facilitating physical adaptations [1]. This strategy enables coaches and practitioners to better plan, adjust, and assess a team’s training to enhance performance, operating under the belief that a blend of training stimuli and ample recuperation will enhance adaptation to training and improve physical fitness and performance [2, 3]. On the contrary, inadequate training or insufficient recovery can potentially lead to an increased risk of injury/illness and a decline in physical readiness [4, 5]. To mitigate these negative consequences, it is crucial to monitor players’ training/match load to inform the programming and adaptation of training and recovery processes [6].

Regarding the comparison of different divisions, previous research analyzed the match running performance among the FA Premier League, now the English Premier League (EPL), English Championship (second division, ECL), and League One [7]. This research found that the players in the first division (Premier League) covered less total distance and had lower high-intensity running distances than those in the lower leagues (ECL and League One). Another similar study compared the EPL and ECL and found that players of the second division covered more total distance, high-intensity running distance and sprint-intensity actions than players of the first division [8]. However, contrasting results were found when examining data from the first and second divisions of Spain, where first division teams covered more total, high-intensity and very high-intensity distances than second division teams [9, 10]. Similarly, another study examining Norwegian football showed higher total and high-intensity distances in first league teams when compared with lower divisions [11]. Thus, the recent studies (≥ 2019) support higher values for first division teams of Spain and Norway, while older research showed the opposite. Therefore, considering data from elite English teams, un update is warranted.

Given the distinct physiological demands of each playing position, external load measures also exhibit variations across different playing positions over a competitive season. In the existing literature, the influence of mediating factors on load, such as playing position, has been thoroughly assessed in the context of professional soccer in the EPL and Spanish First League (LaLiga) [12, 13]. For instance, recent studies have observed that central midfielders, in contrast to attackers and defenders, tend to cover greater total distances at low and medium intensities, as well as moderate-intensity acceleration distances, among elite EPL [14] and Spanish First Division [15] soccer players. Additionally, the available evidence specifically highlighted that wide attackers and wide defenders have shown the highest performance in terms of very high-speed running, high-intensity acceleration, and sprinting distances due to the perpetual attacking and defensive functions in the EPL [14]. Furthermore, in a study assessing the position-specific development of physical performance parameters over a seven-season period in the EPL, it was found that wide and forward positions increased the distance covered at high-intensity and in sprinting more than central defenders and central midfielders [16].

Moreover, it is relevant to highlight that competitive match-play is a dominant component of the physical load completed by soccer players in a training microcycle [17] and constitutes the most important weekly session. Thus, when planning training sessions, performance improvements and lowering injury risk should be major factors, while reference values of match data from various leagues can support this process [18]. In addition, training prescription must consider the level [19] and different playing positions [20].

Given the diverse coaching philosophies ingrained in contemporary elite soccer, it becomes evident that additional research is necessary to enrich our understanding of how training workloads in soccer are structured throughout seasonal cycles or consecutive seasons [13, 19]. Thus, the aims of this study were to: compar training sessions between the EPL and ECL and examine differences between playing positions. The study hypothesis was that the EPL will present higher values than the ECL during training [19].

## MATERIALS AND METHODS

### Study design

This research employed a rigorous four-year longitudinal study design to investigate a male professional team. The study team competed in the EPL and ECL during the study period. The EPL comprises of 38 matches, 19 home and 19 away across a 10-month season, commencing in August and completing in May. While the ECL consists of 46 matches, 23 home and 23 away, across the same duration and calendar period. The study team was promoted from the ECL at the end of season 2020–21, thus the data examined consisted of two ECL seasons and two seasons from the EPL.

A non-probabilistic sampling strategy was adopted to select the participants. The focus of the study was on meticulously monitoring the training load of players during all training sessions. Throughout the entire observation period, spanning from 2019-20 to 2022-23, consistent player monitoring strategies were implemented without any intervention or interference from the researchers in the team’s training processes.

### Participants

Data from 46 1^st^ team outfield soccer players (age 24.6 ± 5.9 years, weight 74.6 ± 7.8 kg, height 1.79 ± 0.09 m) from an English professional club during the complete seasons 2019-20 to 2022-23 seasons were included. The inclusion criteria for the study have been previously applied [19] and included: (i) listed on the roster of the 1^st^ team squad of the English club at the start of each study season, (ii) trained regularly, (iii) participated in at least 80% of training sessions and matches, (iv) did not use dietary supplements during the study, and (v) did not participate in another training program along with this study. Additionally, the exclusion criteria for the study have also been previously employed [19] and included: (i) long-term (three months or longer) injury, (ii) joining the team late in any of the study seasons, (iii) goalkeepers, due to the different variations in the physical demands with outfield players [21].

Players were assigned to a specific position as running demands for these differ significantly. The methodology to differentiate specialized positions was adapted from previous research [22]. As various situational factors have an influence on the style of play that can be modulated by different tactical roles [23], context was considered whilst using a player’s average position in an attempt to determine a player’s relevant tactical role in the team [24]. All participants examined were classified based on their regular playing position at the start of each season and remained consistent throughout each study season: centre-backs (n = 13), full-backs (n = 6), centre midfielders (n = 15), attacking midfielders (n = 8), and centre forwards (n = 4). All data collected resulted from normal player monitoring procedures, nevertheless, written informed consent was obtained from all participants. The study was conducted according to the requirements of the Declaration of Helsinki and was approved by the local Ethics Committee of Cardiff Metropolitan University and the English professional club from which the participants volunteered [25]. To ensure confidentiality, all data were anonymized prior to analysis.

### Training data

Training data were collected over a four-year consecutive period from the 2019–20 to the 2022–23 competitive seasons. Only team pitch-based training sessions were included for analysis. All other sessions, individual training sessions, recovery sessions, and rehabilitation training sessions were excluded [19, 26]. The planning of all soccer content was cyclical in nature and reflective of modern methods of periodization in elite soccer and thus the external physical load experienced by players was undulating across a micro-cycle leading to match-play. The number of days between matches differed [19, 27] and training sessions in elite soccer micro-cycles have recently been classified based on days prior to a match (MD minus (-)) or post-match (MD plus (+)) [12, 19]. All training sessions were integrated to include technical, tactical, physical and mental components. All players completed one or two strength and power gym-based sessions per micro-cycle incorporating upper and lower body and core exercises, although these sessions were not included in the analyses as mentioned earlier [19, 26]. All running training data was collected at the club’s official training facility.

Players only participated in official competitive league matches during a micro-cycle and thus the structure of the training days was standardized across all seasons. The first day post-match (MD+1) were generally a day off and therefore no GPS data was available. Additional fitness sessions for non-starters were limited to the immediate post-match period and GPS data was collected but not included in the study analysis. The start of the next MD micro-cycle was MD-5, five days prior to competition, and targeted compensation training for the non-starters from the previous match and on-field recovery for the starting players. Four days pre-match (MD-4) focussed on drills designed to develop players’ strength, power and ability to repeatedly produce explosive actions. This session was devised to improve technical and tactical understanding when ‘out-of-possession’ whilst developing the necessary physical qualities to produce high accelerations and decelerations without decrement. Individual and unit (defence, midfield, attack) practices followed by positional games and small-sided games with goalkeepers in restricted pitch dimensions were delivered. When delivered, three days prematch (MD-3) aimed to tactically prepare players when ‘in-possession’ whilst developing position-specific high-intensity and sprint running capabilities. Practices entailed full-pitch attacking tactical patterns (10 v 0, 10 v 4) and large numbered games regularly concluding in 11 v 11 format (> 8 v 8 plus goalkeepers). The structure of MD-2, two days prior to the match, concentrated on repeating technical-tactical information at low-intensity in various functional pitch areas and dimensions and thus was regarded as an ‘underloaded’ session considering all key GPS metrics. This session included position-specific passing patterns and then divided players into unit-specific drills for defending or attacking. The final session of the weekly micro-cycle, MD-1, was standardized with no variety and drills intended to provide neural stimulation to players whilst also finalizing tactical situations and set-plays. In micro-cycles where two matches were played (i.e. Saturday and Tuesday), the micro-cycle structure altered to the following: MD+1 off-feet recovery for starting players and compensation training for the non-starters; MD+2 would replicate a standardized MD-1 without any explosive actions (shooting, short sprints). For the purposes of this study, the tactical periodization approach and subsequent training load from all MD-5, MD-4, MD-3, MD-2, and MD-1 training sessions performed across the 2019-20 and 2022-23 seasons were examined. For study reliability and validity, only data from players who performed the full session have been included, withdrawing data from players whose training load was manipulated due to fatigue management or injury [19]. A total number of 840 team training days and 65,219 training data points, that were drills performed by players within training sessions, were examined.

### Data Collection

Physical data were consistently monitored across four study seasons during all training sessions using a 18 Hz Global Positioning System (GPS) technology tracking system (Apex Pod, version 4.03, 50 gr, 88 × 33 mm; Statsports; Northern Ireland, UK) that has been previously validated in a student population for tracking distance covered and peak velocity during simulated team sports and linear sprinting [28]. All devices were activated 30-minutes before data collection to allow the acquisition of satellite signals and to synchronize the GPS clock with the satellite’s atomic clock [29]. Quantifying the devices’ accuracy indicated a 2.5% estimation error in distance covered, with accuracy improving as the distance covered increased and the speed of movement decreased [30]. To avoid inter-unit error, each player wore the same device during the study period [31], although the present GPS system has previously reported excellent inter-unit reliability [32]. Specifically designed vests were used to hold the devices, located on the player’s upper torso, and anatomically adjusted to each player, as previously described [33]. The GPS signal quality and horizontal dilution of position was connected to a mean number of 21 ± 3 satellites, range 18–23, while HDOP across all seasons was 1.3. On completion of each session, GPS data were extracted using proprietary software (Apex, 10 Hz version 4.3.8, Statsports Software; Northern Ireland, UK) as software-derived data is a more simple and efficient way for practitioners to obtain data in an applied environment, with no differences reported between processing methods (software-derived to raw processed) [34]. The dwell time (minimum effort duration) was set at 0.5 s to detect high-intensity running and 1 s to detect sprint distance efforts, in-line with manufacturers recommendations and default settings to maintain consistent data processing [35]. Furthermore, the internal processing of the GPS units utilized the Doppler shift method to calculate both distance and velocity data which is shown to display a higher level of precision and less error compared with data calculated via positional differentiation [35].

Relative distances covered per minute (m/min) in the following categories: total distance (m), high-speed running (HSR) distance (m; total distance covered 5.5–7 m/s); sprint distance (m; total distance covered > 7 m/s); high metabolic load distance (HMLD) (m; the total amount of HSR, coupled with the total distance of accelerations (> 3 m/s^2^) and decelerations (< -3 m/s^2^)) were examined and have been reported based on previous studies [36]. The HMLD variable refers to the distance covered with a power consumption above 25.5 W/kg. This value corresponds to running at a constant velocity of 5.5 m/s or 19.8 km/h on grass. The number of HML efforts (number of efforts performed above 25.5 W/kg), sprint efforts (total number of sprints performed > 7 m/s), accelerations (> 3 m/s^2^ with minimum duration of 0.5 s) and decelerations (< -3 m/s^2^ with minimum duration of 0.5 s) were also examined.

### Statistical Analysis

Descriptive data (mean ± SD) were determined for all GPS variables of interest for position (centre-backs, full-backs, centre midfielders, attacking midfielders, and centre forwards) and league (EPL, ECL). Homogeneity of variance was assessed via Levene’s statistic and, where violated, Welch’s adjustment was used to correct the F-ratio. Multiple two-way (5 × 2) analysis of variance (ANOVA’s) were conducted across all GPS variables to determine the interaction effects between position and league. Post-hoc analysis, using either Bonferroni or Games-Howell post-hoc analyses, where equal variances were and were not assumed, was conducted to identify the differences in training demands between leagues for each position.

Effect size (*η*^2^) values and Cohen’s *d* values (*d*) are also reported for significant results. *η*^2^ values in the range 0–0.0099 are considered insignificant effect sizes, 0.0100–0.0588 as small effect sizes, 0.0589–0.1379 as medium effect sizes, and values greater than 0.1379 as large effect sizes. Cohens *d* effect size magnitudes were interpreted using the following classifications: trivial < 0.19; small 0.2–0.59; 0.6–1.19 moderate; 1.2–1.9 large; 2.0–3.9 very large; > 4.0 extremely large [37]. All significance values were accepted at *p* < 0.05 and all statistical procedures were conducted using JASP (version 0.18) for Macintosh.

## RESULTS

**FIG. 1:**
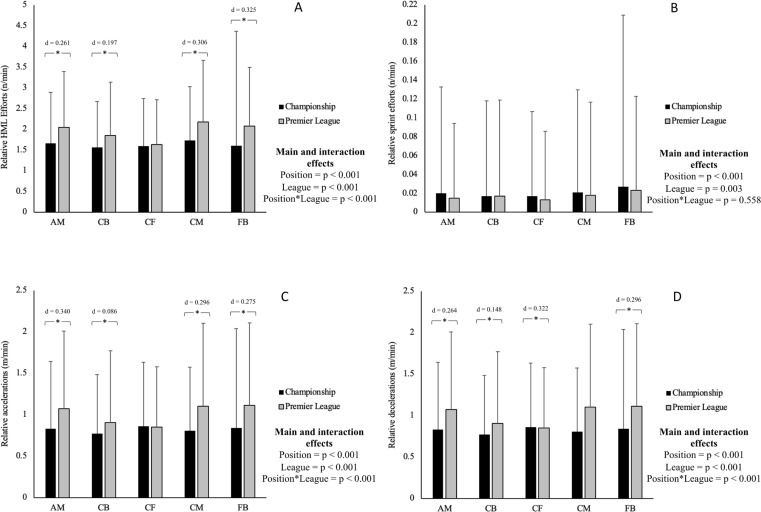
Descriptive statistics for relative explosive efforts per minute for each position in training sessions during English Premier League compared to Championship seasons (mean ± SD). Note: A: HML Efforts per minute; B: Sprint efforts per minute; C: Accelerations per minute; D: Decelerations per minute; *Indicates significant differences between leagues (*p* < 0.05); HML: High metabolic load.

Results of the two-way ANOVA for each GPS metric are reported in Table 1 (mean ± SD) and Figure 1. There was a significant interaction effect between position and league for all GPS metrics (*p* < 0.001; *η*^2^ = 0.001–0.003), except for relative HSR distance, sprint distance, and sprint efforts (*p* > 0.05). Centre-backs and attacking midfielders covered more total distance, HSR distance, and HMLD per minute, and completed more HML efforts, accelerations, and decelerations per minute in training sessions during the EPL compared to the ECL (*p* < 0.001–0.002; *d* = 0.086–0.340). Centre midfielders and full-backs completed more total distance and HMLD per minute, and completed more HML efforts, accelerations, and decelerations per minute in EPL training compared to the ECL (*p* < 0.001; *d* = 0.164–0.325). Finally, centre forwards covered more total distance per minute in training sessions in the EPL compared to the ECL (*p* = 0.017; *d* = 0.089).

**TABLE 1:** Descriptive statistics for relative distances covered for each position in training sessions during the English Premier League compared to English Championship seasons.

		Premier League	Championship	Effect Size (Cohen's d)	Significance (p value)	Main and Interaction Effects	
Relative Distance (m/min)	CB	81.84 ± 33.93	74.14 ± 32.72	0.167	< 0.001	Position	p < 0.001
	FB	85.80 ± 34.35	78.25 ± 34.76	0.164	< 0.001	League	p < 0.001
	CM	88.86 ± 36.28	78.90 ± 38.86	0.216	< 0.001		
	AM	84.37 ± 33.56	75.87 ± 34.48	0.185	< 0.001	Position*League	p < 0.001
	CF	77.59 ± 31.98	74.78 ± 32.95	0.089	0.017		

Relative HSR Distance (m/min)	CB	5.05 ± 14.25	3.82 ± 11.02	0.097	< 0.001	Position	p < 0.001
	FB	6.00 ± 12.74	5.48 ± 17.25	0.040	1.000	League	p < 0.001
	CM	5.17 ± 12.12	4.62 ± 5.17	0.044	0.260		
	AM	5.76 ± 12.44	4.67 ± 10.84	0.086	0.002	Position*League	p = 0.138
	CF	4.94 ± 11.99	4.47 ± 11.15	0.038	1.000		

Relative HMLD (m/min)	CB	16.87 ± 15.71	14.00 ± 13.61	0.176	< 0.001	Position	p < 0.001
	FB	19.36 ± 15.74	15.58 ± 19.64	0.232	< 0.001	League	p < 0.001
	CM	19.56 ± 15.28	15.81 ± 19.56	0.230	< 0.001		
	AM	19.04 ± 14.98	15.51 ± 14.13	0.216	< 0.001	Position*League	p < 0.001
	CF	15.56 ± 13.76	14.81 ± 14.11	0.046	1.000		

Relative Sprint Distance (m/min)	CB	0.36 ± 2.45	0.40 ± 2.73	-0.016	1.000	Position	p < 0.001
	FB	0.44 ± 2.25	0.59 ± 3.94	-0.058	0.588	League	p < 0.001
	CM	0.34 ± 2.22	0.46 ± 0.34	-0.047	0.149		
	AM	0.29 ± 1.80	0.44 ± 3.14	-0.060	0.207	Position*League	p = 0.588
	CF	0.25 ± 1.74	0.34 ± 2.32	-0.035	1.000		

Note: CB = centre-back; FB = full-back; CM = centre midfielder; AM = attacking midfielder; CF = centre forward; HSR = high-speed running; HMLD = high metabolic load distance

There was also a significant main effect for league for all GPS metrics (*p* < 0.001; *η*^2^ = 0.001–0.009), with EPL training sessions resulting in greater total distance per minute, HSR distance per minute, HMLD per minute, and number of HML efforts, accelerations, and decelerations per minute compared to training in the ECL (*p* < 0.001; *d* = 0.061–0.224). Sprint distance per minute and the number of sprints per minute were higher in the ECL training sessions compared to the EPL (*p* < 0.001, *d* = 0.043; *p* = 0.003, *d* = 0.031, respectively).

Findings revealed a significant main effect for position for all GPS metrics (*p* < 0.001; *η*^2^ = 0.001–0.005). Post-Hoc analysis confirmed that centre midfielders covered more distance per minute than all other positions (*p* < 0.001, *d* = 0.040–0.167), while full-backs and attacking midfielders also covered more than centre-backs and centre forwards (*p* < 0.001–0.018, *d* = 0.041–0.127). For HSR per minute, full-backs covered more than centre-backs, centre midfielders and centre forwards (*p* < 0.001, *d* = 0.067–0.103), while attacking midfielders also covered more than centre-backs (*p* < 0.001, *d* = 0.061). Attacking midfielders, centre midfielders, and full-backs covered more HMLD per minute than centre-backs and centre forwards (*p* < 0.001, *d* = 0.113–0.153). Attacking midfielders, centre midfielders, and full-backs covered more HML efforts per minute than centre-backs and centre forwards (*p* < 0.001–0.013, *d* = 0.061–0.229). Full-backs covered more sprint distance per minute and completed more sprints per minute than all other positions (*p* < 0.001, *d* = 0.054–0.098). Attacking midfielders, centre midfielders and full-backs covered more accelerations and decelerations per minute than centre forwards (*p* < 0.001–0.003, *d* = 0.053–0.134).

## DISCUSSION

The main findings from the present study showed higher training values in the EPL compared to the ECL with the exception of sprint (both in distance and efforts) that showed higher training values in the ECL (sig-nificant differences, although effect sizes were trivial). When comparing player positions, loading pattern varied between metrics.

The results of the present study showed higher values during the EPL season compared with the ECL season, which contradicts older research in an English team [7, 8] but is in line with previous studies that examined first and second division Spanish [9, 10] and Norweigan [11] teams and found higher load demands in first division teams. This may partly be attributed to the first division league requiring a higher physical capacity and consequently, higher match running capacity [38]. Another explanation may be related to the playing formation implemented by the first division teams that may require higher external loads [39], although this variable was not addressed in the present study. However, previous studies [7–11] analyzed match data that was not examined in the present study that only included training data. Nonetheless, considering that match-play is regarded as the most important session of the training week with the highest load [17], training session design should understand and utilize match data values as a reference. Thus, higher training loads would be expected in the EPL when compared with the ECL [20].

In addition, recent research compared senior (first team) and U-18 soccer players from the same EPL team and reported higher high-intensity (5.5–7 m/s) and sprint (> 7 m/s) values for first team players compared with U-18 [19]. Furthermore, U-18 players covered higher total distance than first team players that may be associated with the lower competitive level of the U-18 players [19]. However, contrasting results were found in a recent study that compared first and U-18 soccer players from the same Scottish Premier team and found no differences in external load measures between groups [18]. Although, these studies had different designs to the present research and U-18 soccer players were examined, while the current study investigated the same senior (first team) players competing in the EPL and ECL (> 18 years) [18, 19].

As previously mentioned, a minor exception was found in sprinting, both in distance and efforts. However, it should be acknowledged that differences in sprint distance, efforts and relative distances were trivial, although statistically significant. This may partly be explained by the very large number of drills examined. Moreover, in both leagues there was a very small amount of sprint distance (0.3–0.4 m/min) and sprint efforts (0.02 efforts/min) during the examined training sessions (see Figure 1B). This may possibly be linked to sprint distance being equal to zero in drills, and thus can also be highlighted as a limitation. Furthermore, indeed style of play and team formation were not considered in the present study and recently a study that examined EPL players showed that formation and possession can have a significant impact on total distance, HSR, and HMLD [40]. Although these contextual factors lead to speculation that may partly explain the current results, however more research is warranted to confirm this notion.

Considering playing position, the usual trend of higher total distance values for centre midfielders was confirmed. This position was followed by full-backs and attacking midfielders. The same scenario occured for HML distance and efforts. Moreover, full-backs showed higher values for HSR, accelerations and decelerations and were followed by attacking and central midfielders. Full-backs also showed higher values of sprinting, both distance and efforts and were followed by centre-backs and centre midfielders. The present results were similar to those reported in a recent systematic review although some differences were evident. For example, wide midfielders, although not examined in the present study, and centre forwards covered greater running distances (> 14 km/h), while central midfielders performed a higher number of accelerations and decelerations [41]. However, it is relevant to highlight that the playing position findings are associated with the differing tactical roles within the team, particularly when defending and attacking. Specifically, the general trend of this study showed higher values for centre midfielders, full-backs and attacking midfielders that can be associated with covering a larger action zone in both training and matches [42]. Therefore, a fundamental attribute for these positions is a higher aerobic capacity than other positions such as central-backs and centre forwards [43].

### Practical Applications, Limitations and Future Perspectives

Considering practical applications, it may be suggested that the EPL training was more demanding than the ECL with the exception of sprint measures. This information is relevant for ECL coaches and performance staff to obtain knowledge on the training load values performed in the EPL. Similarly, for EPL coaches and staff these findings may support training design to maintain EPL status and avoid relegation to the ECL. Furthermore, sports scientists may utilize the findings of the current study to design position-specific physical conditioning training and individualized recovery sessions [44], whilst considering league standard and position. Finally, to aid practitioners design more effective training, contextualizing key physical demands with tactical structure may be of great benefit.

Despite the findings of the current study, there are some limitations that should be listed. As mentioned, style of play and playing formation were not considered and might possibly explain some of the current study findings. Moreover, the evolution of the team across the four seasons would provide additional knowledge for coaches and performance staff. For instance, it could reveal evolution in terms of external load according to possession classification, playing style and formation [9, 40, 45]. Therefore, the aforementioned variables should be considered for future research. Finally, all data should be cautiously interpreted as only one team from the EPL and ECL was examined, therefore the generalization to different leagues/countries must be considered.

## CONCLUSIONS

The main conclusion was associated with higher training values during the EPL with the exception of sprint, both in distance and efforts. Nonetheless, this study showed the importance of greater demands in HSR and sprint distances of EPL training when compared with ECL sessions. The values presented in this study constitute possible reference values that may be used by coaches, performance staff, or practitioners to achieve desirable competitive levels to cope with EPL and ECL demands. Furthermore, these findings may allow coaches of ECL teams to replicate such values or even increase during specific training sessions in order to prepare players for the EPL. In addition, the present data provides some guidance on the differing physical demands placed on various positions and may support coaches and practitioners to design position-specific drills incorporating physical and technical/tactical strategies. Nevertheless, all presented values should be interpreted with caution since only data from one team was utilized.
